# Clinical implementation of a fully automated quantitative perfusion cardiovascular magnetic resonance imaging workflow with a simplified dual-bolus contrast administration scheme

**DOI:** 10.1038/s41598-024-60503-x

**Published:** 2024-04-26

**Authors:** S. Borodzicz-Jazdzyk, C. E. M. Vink, A. Demirkiran, R. Hoek, G. W. de Mooij, M. B. M. Hofman, A. Wilgenhof, Y. Appelman, M. Benovoy, M. J. W. Götte

**Affiliations:** 1 Department of Cardiology, Amsterdam UMC, Vrije Universiteit Amsterdam, Amsterdam Cardiovascular Sciences, De Boelelaan 1118, 1081 HV Amsterdam, The Netherlands; 2 1st Department of Cardiology, Medical University of Warsaw, Banacha 1a Str., 02-097 Warsaw, Poland; 3 Department of Radiology and Nuclear Medicine, Amsterdam UMC, Vrije Universiteit Amsterdam, De Boelelaan 1118, 1081 HV Amsterdam, The Netherlands; 4 Area19 Medical Inc., Montreal, H2V2X5 Canada

**Keywords:** Coronary artery disease and stable angina, Ischaemia

## Abstract

This study clinically implemented a ready-to-use quantitative perfusion (QP) cardiovascular magnetic resonance (QP CMR) workflow, encompassing a simplified dual-bolus gadolinium-based contrast agent (GBCA) administration scheme and fully automated QP image post-processing. Twenty-five patients with suspected obstructive coronary artery disease (CAD) underwent both adenosine stress perfusion CMR and an invasive coronary angiography or coronary computed tomography angiography. The dual-bolus protocol consisted of a pre-bolus (0.0075 mmol/kg GBCA at 0.5 mmol/ml concentration + 20 ml saline) and a main bolus (0.075 mmol/kg GBCA at 0.5 mmol/ml concentration + 20 ml saline) at an infusion rate of 3 ml/s. The arterial input function curves showed excellent quality. Stress MBF ≤ 1.84 ml/g/min accurately detected obstructive CAD (area under the curve 0.79; 95% Confidence Interval: 0.66 to 0.89). Combined visual assessment of color pixel QP maps and conventional perfusion images yielded a diagnostic accuracy of 84%, sensitivity of 70% and specificity of 93%. The proposed easy-to-use dual-bolus QP CMR workflow provides good image quality and holds promise for high accuracy in diagnosis of obstructive CAD. Implementation of this approach has the potential to serve as an alternative to current methods thus increasing the accessibility to offer high-quality QP CMR imaging by a wide range of CMR laboratories.

## Introduction

Stress perfusion cardiovascular magnetic resonance (CMR) imaging is an established non-invasive method for detection of myocardial ischemia^1^. In daily clinical practice, stress perfusion CMR is evaluated by visual assessment of first-pass perfusion images, where the hypo-perfused myocardial segments show delayed gadolinium-based contrast agent (GBCA) wash-in kinetics during first pass, resulting in areas with lower signal intensity (SI; i.e. perfusion defect). Recent technical advances allow absolute quantification of myocardial blood flow (MBF) by applying CMR perfusion imaging (quantitative perfusion CMR; QP CMR)^2^.

A critical step for MBF quantification is the accurate assessment of the arterial input function (AIF), which reflects the proportion of GBCA entering the coronary circulation. Since myocardial contrast enhancement is considered to have a linear response to arterial contrast enhancement, it is crucial to make sure the relation between GBCA concentration and SI in the blood pool is also linear^3^. However, high concentrations of a GBCA in the blood pool as a result of a standard single-bolus injection (typically 0.05–0.1 mmol/kg) may exacerbate the T1 and T2* saturation effect, which causes a non-linear relation between SI and GBCA concentration. This non-linearity results in a truncated or dampened AIF curve. As a consequence, the MBF may be wrongly overestimated following the deconvolution process, affecting the overall QP CMR result and interpretation^4^. As a solution, two different approaches of AIF saturation correction are available, including the dual-sequence and dual-bolus method. The dual-sequence method uses a single dose bolus of contrast medium and combines two different types of image acquisitions within the same cardiac cycle, i.e. short saturation recovery with low spatial resolution and reduced T1-weighting for AIF, and long saturation recovery with high spatial resolution for myocardial enhancement^5^. In general, the dual-sequence approach is considered more easy to use with a single bolus of GBCA and simultaneous assessment of AIF and myocardial SI. However, its major limitation is the requirement of a specific, dedicated pulse sequence^6^. The dual-bolus approach enables the use of high dose of GBCA for myocardial perfusion analysis (typically 0.05–0.1 mmol/kg) combined with measurement of the AIF using an initial low-dose bolus (typically 0.0025–0.005 mmol/kg, ~ 1:10 concentration of the main bolus) of contrast injection, which preserves the linearity of SI to GBCA concentration in the blood pool and maintains high contrast-to-noise ratio. In this manner, the AIF can be accurately determined and is less affected by the substantial T1 and T2* saturation effect^4,7^. In contrast to dual-sequence, the dual-bolus method uses standard, regulatory-approved sequences for first-pass perfusion imaging, which makes it more widely available and easy to implement for general CMR centers. However, in a standard approach, the dual-bolus GBCA administration scheme requires an equal volume of both the main bolus of neat GBCA and the pre-bolus^7^. This requires a time-consuming preparation of the pre-bolus which is considered the major limitation of this technique, due to its inconvenience in integrating into daily clinical workflow. Nevertheless, simplified protocols with a non-diluted pre-bolus of small volume have been successfully implemented^8–10^.

Until recently, QP CMR required cumbersome and time-consuming operator-dependent analysis, which made it prone to substantial interobserver variability and prevented its widespread use in clinical routine^11–25^. To address these limitations, the fully automated pixel-wise QP CMR image processing framework has been established, which is reproducible, time-efficient and does not require manual delineation of regions of interests (ROIs) or myocardial image segmentation^12,17,19,20,26–28^.

In this study, we clinically implemented a ready-to-use QP CMR workflow with a simplified dual-bolus GBCA administration scheme and fully automated QP post-processing. The study aimed to evaluate (1) the image quality, and (2) results and diagnostic accuracy of the proposed QP CMR workflow, to identify obstructive coronary artery disease (CAD) in patients with suspected CAD who underwent adenosine stress perfusion CMR.

## Methods

The study retrospectively analyzed 25 patients with suspected myocardial ischemia who underwent both adenosine stress perfusion CMR imaging with the simplified dual-bolus scanning protocol and invasive coronary angiography (ICA) or coronary computed tomography angiography (CCTA) as a clinical routine at Amsterdam UMC, location VUmc.

ICA was used to confirm or exclude presence of obstructive CAD, while CCTA was used only to exclude obstructive CAD. Obstructive CAD was defined as ≥ 70% stenosis in at least one major coronary vessel or > 50% stenosis in the left main coronary artery by visual assessment of the coronary angiogram, or fractional flow reserve (FFR) ≤ 0.8. Non-obstructive CAD was defined as: (1) < 70% stenosis in ICA, or (2) FFR > 0.8, or (3) < 30% stenosis and a calcium score 0 in all major coronary arteries in CCTA. Performance of FFR during ICA was on the discretion of the operator, and according to the currently available guidelines and recommendations. The study was approved by the Medical Ethics Review Committee of the Amsterdam UMC, location VUmc. All methods were performed in accordance with the relevant guidelines and regulations. Informed consents from all participants were obtained.

### CMR image acquisition

Images were acquired using a 3 T whole-body MR scanner (Magnetom Vida, Siemens Healthcare, Erlangen Germany). Patients were instructed to avoid products containing caffeine or xanthine for at least 24 h before the examination.

### Simplified dual-bolus protocol

**Figure 1:**
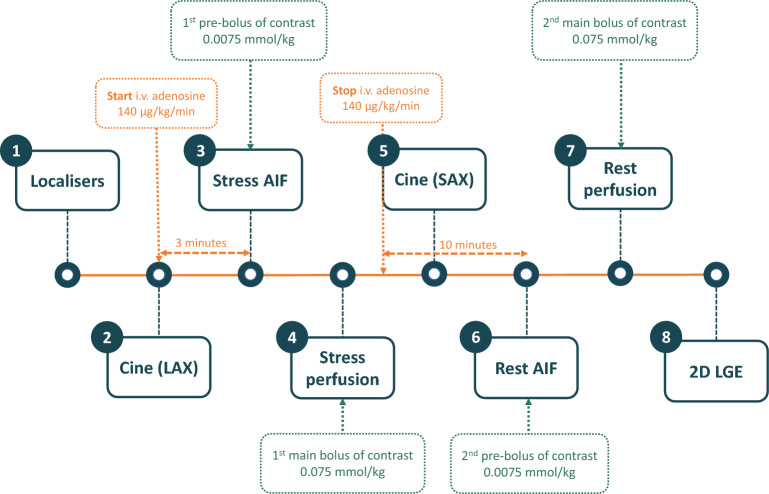
Proposed adenosine stress perfusion dual-bolus CMR scanning protocol. *AIF* arterial input function.

The scanning protocol with simplified dual-bolus contrast administration scheme is presented in Fig. 1. Stress AIF and perfusion images were acquired after at least 3 min of constant intravenous infusion of adenosine (140 μg/kg/min) while patients held their breath for as long as possible. AIF images were obtained during intravenous administration of a non-diluted pre-bolus of GBCA (DOTAREM^®^, Guerbet, Villepinte, France 0.5 mmol/ml) at a dose of 0.0075 mmol/kg, infusion rate 3 ml/s, flushed with 20 ml of saline. Perfusion images were obtained using a non-diluted main bolus of GBCA (DOTAREM^®^, Guerbet, Villepinte, France 0.5 mmol/ml) at a dose of 0.075 mmol/kg, infusion rate 3 ml/s, flushed with 20 ml of saline. GBCA and saline were administered intravenously using an MRI-compatible power injector. Notably, the pre-bolus to bolus concentration ratio was 1:10, as required for the dual-bolus approach^7^. Perfusion images were acquired using a commercially available saturation recovery turbo spoiled gradient-echo sequence at every heartbeat over at least 50 beats in three parallel short-axis slices at the basal, mid-ventricular and apical levels. At the beginning of each acquisition, two proton density-weighted images were obtained at all three levels for correcting surface-coil SI inhomogeneity. Typical in-plane resolution of the myocardial perfusion images was 2.25 × 2.25 mm^2^, with a slice thickness of 8 mm (repetition time 2.64 ms, echo time 1.18 ms, inversion time 125 ms, flip angle 12^°^, matrix size 160 × 120), and a twofold acceleration using GRAPPA/T-pat. The temporal acquisition window for the perfusion images was 158 ms. Rest AIF and rest perfusion images were acquired using identical pre-bolus and bolus dosages and slice location, at least 10 min after finishing adenosine administration. In case heart rates exceeding 90 bpm, each set of perfusion images was acquired over two heartbeats. No raw data filtering was performed.

### CMR image post-processing and analysis

QP CMR analysis was performed using cvi42 software (prototype version 5.13.8.2774, Circle Cardiovascular Imaging Inc., Calgary, Canada) equipped with a newly updated, fully automated pixel-wise QP module, described in detail elsewhere^19^. Briefly, the module automatically corrected in-plane respiratory motion of the heart and the surface coil-induced signal inhomogeneities using the proton density images^26,29^. Next, the AIF and myocardial ROIs were detected to derive time-SI curves and identify key time points during first-pass contrast enhancement^28^. The basis for MBF quantification was based on the central volume principle described by Zierler for indicator-dilution experiments, where the SI observed in the myocardium at each pixel is modeled as the convolution of the AIF and a residual function, for which its amplitude is the scalar MBF value. The MBF at each pixel can thus be derived through a reverse process of deconvolution of a pixel’s myocardium SI curves and the AIF curve^3,12,19,30^. Inappropriate automatic delineation of myocardial of AIF ROIs was corrected by the operator and care was taken to prevent inclusion of blood pool into myocardial ROIs.

### Assessment of image quality and diagnostic accuracy

First, blinded to ICA and FFR results, the two independent CMR level 3 experts (M.G. > 10 years of experience, A.D. > 6 years of experience) were asked to allocate each case to the obstructive or no obstructive CAD group based on the QP CMR and LGE images. Using the color pixel QP maps, image readers were instructed to diagnose ischemia if two adjacent segments within a coronary territory each demonstrated stress MBF below the established study-specific cut-off value in the absence of LGE. Subsequently, the imaging experts viewed the conventional gray-scale first-pass perfusion images and assessed the image quality using a 5-point Likert scale, including assessment of artifacts, noise, overall impression of the image quality and certainty in diagnosis (Supplementary Fig. S1). Finally, after viewing the gray-scale perfusion images, the image readers were asked again to classify the patient into the obstructive CAD or no obstructive CAD group. For the assessment of diagnostic accuracy of QP and gray-scale images, in cases of discordance between the two readers regarding diagnosis (obstructive CAD or no obstructive CAD), a third image reading expert experienced in QP analysis (S.B.J.) evaluated the scan and this assessment was used for the analysis.

### Statistical analysis

Statistical analysis was performed with the SPSS software package (IBM SPSS Statistics 20.0, Chicago, IL, USA) and MedCalc (MedCalc Software 12.7.8.0, Mariakerke, Belgium). Continuous variables are expressed as mean ± standard deviation (SD) for normally distributed data or median with interquartile range (IQR) for non-normally distributed variables. One-way ANOVA was used to compare the MBF estimates between groups. A p-value < 0.05 was considered statistically significant. A receiver operating characteristics (ROC) curve per-vessel analysis was performed to assess the diagnostic accuracy of newly proposed dual-bolus QP workflow for detection of obstructive CAD. The Youden index was used to identify the optimal stress MBF cut-off value on a per-vessel basis using the mean stress MBF value of two lowest adjacent segments within a coronary territory. Coronary territories with presence of LGE were excluded from the analysis. Therefore, the study-specific calculated optimal cut-off value of stress MBF was used by the CMR expert readers to assign the patients into obstructive or no obstructive CAD groups based on QP CMR results.

### Ethics approval and consent to participate

The study was approved by the Medical Ethics Review Committee of the Amsterdam UMC, location VUmc. Informed consents from all participants were obtained.

## Results

**Figure 2:**
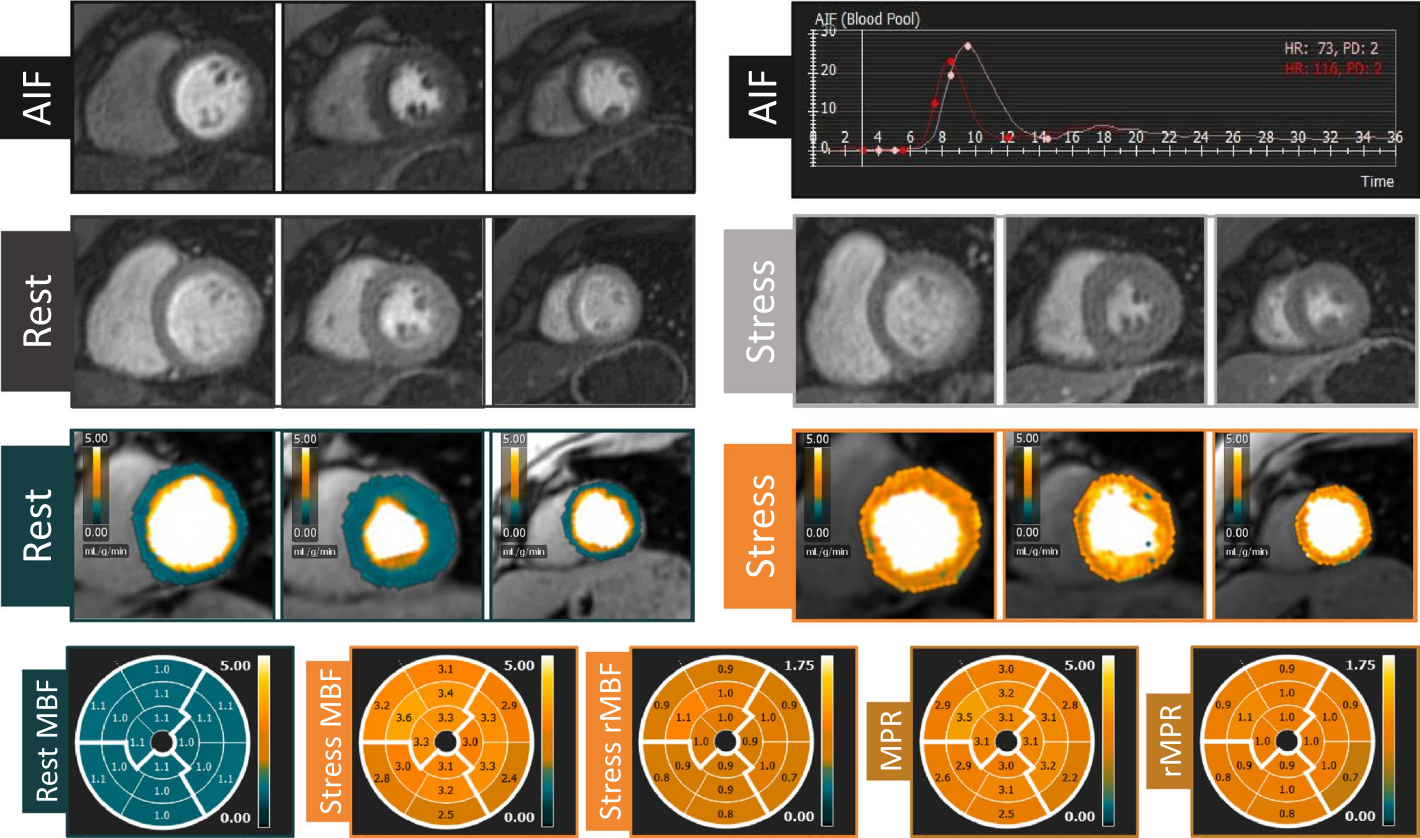
Example of images and quantitative perfusion results in a patient without obstructive coronary artery disease. *AIF* arterial input function, *red curve* stress arterial input function, *white curve* rest arterial input function, *MBF* myocardial blood flow, *MPR* myocardial perfusion reserve, *rMBF* relative myocardial blood flow, *rMPR* relative myocardial perfusion reserve.

**Figure 3:**
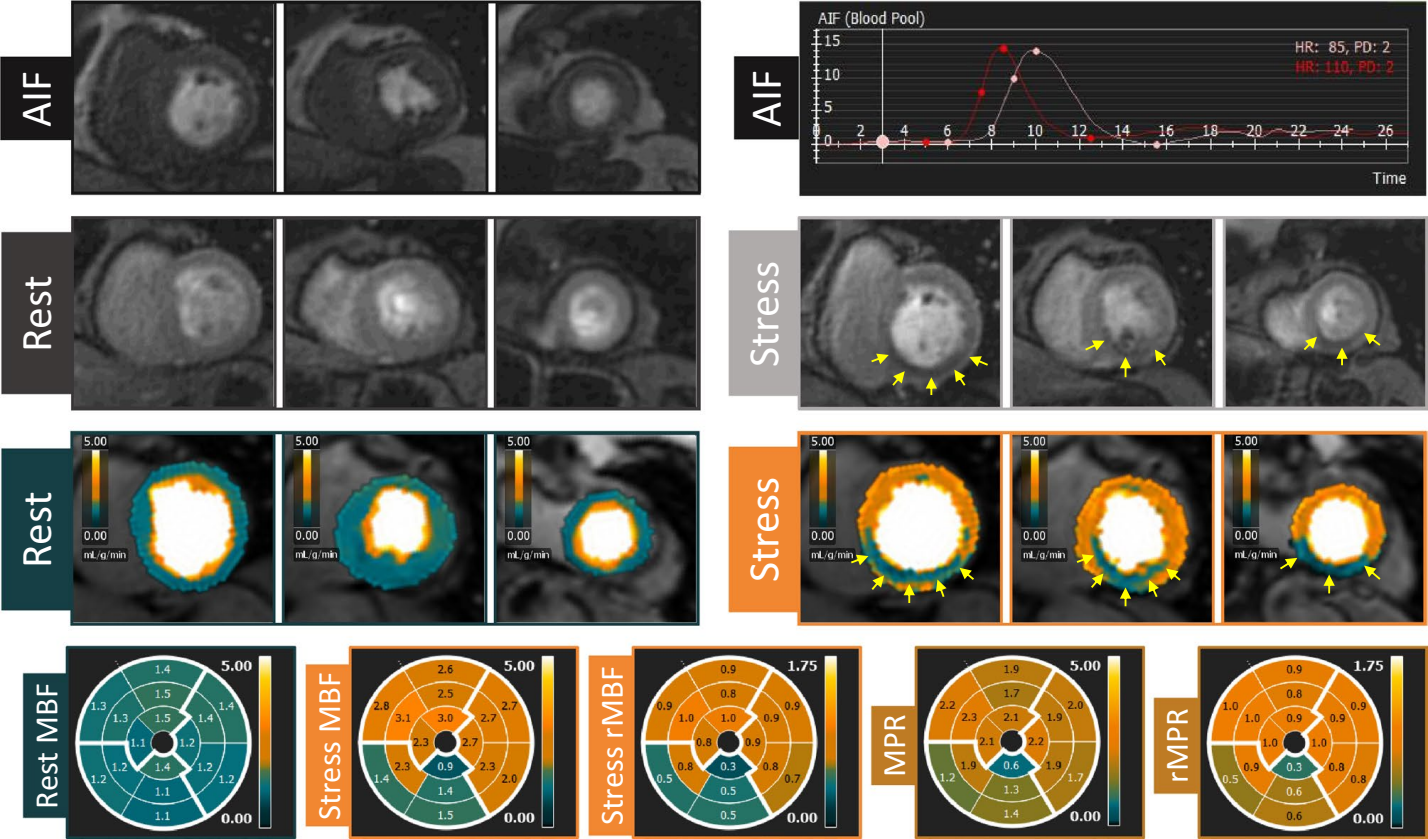
Example of images and quantitative perfusion results in a patient with right coronary artery obstruction. *AIF* arterial input function, *red curve* stress arterial input function, *white curve* rest arterial input function, *MBF* myocardial blood flow, *MPR* myocardial perfusion reserve, *rMBF* relative myocardial blood flow, *rMPR* relative myocardial perfusion reserve. Arrows indicate perfusion defect in right coronary artery territory.

**Figure 4:**
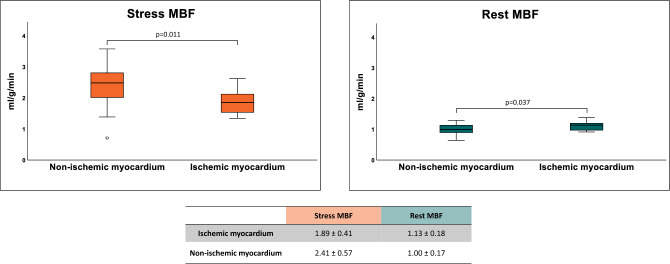
Comparison of stress and rest myocardial blood flow between ischemic and non-ischemic myocardium. *MBF* myocardial blood flow.

Baseline characteristics of the population studied according to the simplified dual-bolus protocol (n = 25) is presented in Table 1. The median age was 60 (IQR 54–71) years, and 64% of patients were male. All patients had sufficient response to adenosine based on the analysis of stress MBF, presence of splenic switch-off and heart rate increase (average heart rate increase from 66 ± 12 to 93 ± 15 bpm, p < 0.01) during stress perfusion. In total 23 patients underwent ICA, whereas 2 patients underwent CCTA (which was used only to exclude obstructive CAD). After exclusion of coronary territories with LGE (n = 19), in total 56 vessels were analyzed (18 left anterior descending arteries (LAD; 32%), 18 right coronary arteries (RCA; 32%) and 20 circumflex arteries (Cx; 36%). Based on ICA and/or FFR, obstructive CAD was found in 10 (40%) patients including 6 patients with single vessel disease and 4 patients with 2-vessel disease. FFR was determined in a minority of vessels (7/56; 5 LAD, 1 Cx and 1 RCA) whereas 3 vessels had a critical stenosis (> 95% of luminal narrowing). Significant coronary artery obstruction was found in 9 coronary arteries, including 4 LAD (22%), 2 RCA (11%) and 3 Cx (15%). Typical examples of acquired images, AIF stress and rest curves, and results of the QP CMR analysis in patients without and with obstructive CAD are shown in Figs. 2 and 3, respectively. In a patient without obstructive CAD, no perfusion defect was visible on both the conventional gray-scale first-pass perfusion images and QP CMR results, and the average stress MBF value was 3.1 ml/g/min (Fig. 2). In a patient with obstructed RCA, both the conventional first-pass perfusion images and QP CMR results show a perfusion defect and reduced stress MBF, respectively, in the corresponding RCA perfusion territory segments. In the hypo-perfused, ischemic segments, the average stress MBF value was 1.3 ml/g/min, whereas the average MBF in the remote myocardium was 2.6 ml/g/min. In the total population, the mean stress MBF was significantly lower in myocardial territories supplied by a vessel with a significant obstruction (ischemic myocardium) when compared to regions supplied by a vessel with no significant lesion (non-ischemic myocardium; 1.89 ± 0.41 vs. 2.41 ± 0.57 ml/g/min, p = 0.01; Fig. 4). The mean rest MBF in ischemic myocardium was 1.13 ± 0.18 ml/g/min, whereas in non-ischemic myocardium the mean value was 1.00 ± 0.17 ml/g/min (p = 0.04; Fig. 4).

**Table 1:** Baseline characteristics of study population.

Age (years)	60 (IQR 54–71)
Male	16 (64%)
Body mass index (kg/m^2^)	26 ± 4
History of coronary artery disease	10 (40%)
PCI	7 (28%)
MI	4 (16%)
Risk factors	
Family history of CAD	9/23 (39%)
Hypertension	11 (44%)
Dyslipidemia	10 (40%)
Diabetes mellitus	4 (16%)
Smoking	13 (52%)
Medication	
ACE inhibitor or ARB	9 (36%)
Aspirin	18 (72%)
Beta-blockers	14 (56%)
Calcium channel blockers	8 (32%)
Nitrates	10 (40%)
Statin	18 (72%)

*ACE* angiotensin converting enzyme, *ARB* angiotensin receptor blockers, *CAD* coronary artery disease, *IQR* interquartile range, *MI* myocardial infarction, *PCI* percutaneous coronary intervention.

**Figure 5:**
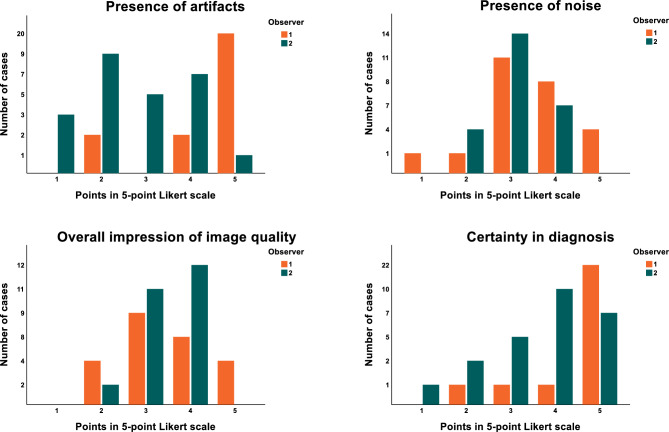
Results of qualitative assessment of image quality by two independent level 3 CMR experts.

Table 2 and Fig. 5 show results of expert assessment of image quality by two independent level 3 CMR imaging readers. The overall impression of the quality of the majority of cases was assessed as at least moderate by both readers. Moreover, the observers graded the diagnostic confidence as good–excellent (4–5 points in Likert scale) in the majority of cases (92% for observer 1, 68% for observer 2) (Table 2, Fig. 5).

**Table 2:** Image quality assessment of the dual-bolus protocol.

Measure	Mean value
Presence of artifacts	3.7 ± 0.7
Presence of noise	3.3 ± 0.6
Overall impression of image quality	3.4 ± 0.6
Certainty in diagnosis	4.3 ± 0.6

### Diagnostic accuracy of QP CMR workflow

ROC analysis showed the optimal cut-off value for regional stress MBF ≤ 1.84 ml/g/min to predict significant coronary artery obstruction with a sensitivity of 89% [95% Confidence Interval (CI): 52% to 100%)], specificity of 70% [95% CI 55% to 83%], positive predictive value (PPV) of 36% [95% CI 26% to 48%] and negative predictive value (NPV) of 97% [95% CI 84% to 100%]. Area under the curve (AUC) was 0.79 [95% CI 0.66 to 0.89].

Visual assessment of only color pixel QP maps by CMR experts yielded an accuracy of 80% [95% CI 59 to 93%], with sensitivity of 90% [95% CI 56% to 100%] and specificity of 73% [95% CI 45% to 92%], PPV of 69% [95% CI 39% to 91%], NPV of 92% [95% CI 62% to 100%]. In addition, combined visual analysis of both color pixel QP maps and conventional gray-scale first-pass perfusion images yielded an even higher diagnostic accuracy of 84% [95% CI 64% to 95%]; with sensitivity of 70% [95% CI 35% to 93%] and specificity of 93% [95% CI 68% to 100%], PPV of 88% [95% CI 47% to 100%] and NPV of 82% [95% CI 57% to 96%].

## Discussion

This study clinically implemented a ready-to-use QP CMR workflow with a simplified dual-bolus GBCA administration scheme and fully automated QP image post-processing. The results demonstrated, that this workflow provides good image quality and holds promise for high diagnostic accuracy for detecting obstructive CAD. The proposed approach is obtainable, easy to implement and only requires a commercially available MRI pulse-sequence and post-processing software. Therefore, it may facilitate the introduction of QP CMR in general CMR centers and offers potential to improve the clinical performance of centers already performing qualitative CMR perfusion imaging^23,31,32^. This easy-to-implement strategy may contribute to a more widespread adoption of non-invasive accurate and quantitative assessment of ischemia by CMR.

As described by Ishida et al., a standard dual-bolus GBCA injection scheme for QP CMR requires dilution of pre-bolus to an equal volume as the main bolus of neat GBCA^7^. However, this approach requires additional pre-scan preparation, where the pre-bolus has to be diluted to 10% solution which should be preloaded to the infusion line. This impairs the time efficiency of the CMR laboratory workflow. Several studies successfully used the simplified dual-bolus GBCA administration scheme, where the pre-bolus is in fact a small volume bolus of contrast^8–10^. Such a simplified dual-bolus approach introduced by Köstler et al. used fixed high doses of GBCA (1 ml pre-bolus of Magnevist 0.5 mmol/ml, Schering, Germany, followed by 8 or 12 ml main bolus at an infusion rate 4 ml/s followed by a flush of 20 ml saline)^9^. Others used dual-bolus protocols with a fixed dose of pre-bolus (1 ml of Magnevist 0.5 mmol/ml, Schering, Germany followed by 15 ml saline) and main bolus at a dose of 0.1 mmol/kg followed by 15 ml saline injected at infusion rate 3 ml/s^8^. The strength of approach proposed in the current study, however, is administration of a (relatively) higher dose of pre-bolus GBCA (0.0075 mmol/kg), adjusted to patient weight. As previously shown, higher GBCA dosages increase both signal-to-noise ratio and contrast-to-noise ratio and improve the diagnostic performance of stress perfusion CMR in detection of obstructive CAD, but may be related to the substantial saturation effect which limits the accuracy of QP^4,33,34^.

As the proposed approach uses a non-diluted pre-bolus, the moderate infusion rate at 3 ml/s was chosen as a compromise between the risk of temporary GBCA accumulation at the level of LV cavity and associated T1 and T2* saturation effect, and the risk of possible contrast dispersion within the bloodstream. In QP CMR a crucial factor affecting the accuracy of measurements is the linearity between the SI and the amount of administered GBCA^3,35^. Gebker et al. have shown significant differences in myocardial enhancement in regard to the injection rate, where the rate of 2 ml/s was associated with a significantly lower myocardial enhancement in comparison with 4 ml/s at a GBCA dose of 0.05 mmol/kg^35^. The authors concluded, that administration of GBCA in a prolonged injection with very low injection rates results in substantial dispersion that it cannot achieve an enhancement comparable to a more concentrated bolus at higher injection rates^35^. Similarly, myocardial SI curves showed lower upslope when the GBCA was injected at a rate of 2 ml/s compared to 3 and 4 ml/s. However, with faster injection rates the amount of inter-individual dispersion increases^35^. Therefore, it has been suggested, that perfusion examinations should be performed with injection rates of 3–4 ml/s^35^.

In this study, imaging was performed using a 3 T scanner, what is beneficial for image quality and diagnostic performance of first-pass perfusion imaging for ischemia detection^13^. The proposed simplified dual-bolus protocol applied at 3 T indeed demonstrated relatively good image quality, as assessed by two independent level 3 CMR experts. Although the assessment using 5-point Likert scale is highly subjective and therefore may substantially differ between the expert readers, the majority of cases were assessed having at least moderate overall image quality with good-to-excellent diagnostic confidence.

Most importantly for QP CMR analysis, this workflow obtained excellent quality of acquired AIF curves in the absence of substantial T2* effects, which allows absolute quantification of MBF. Studies have shown, that quantitative estimation of myocardial perfusion may increase the diagnostic performance of CMR in evaluation of presence and severity of obstructive CAD^23,32^. QP CMR may be also a promising non-invasive imaging method for detection of myocardial ischemia in symptomatic patients with suspected coronary microvascular dysfunction (CMD)^6,23,31^. Absolute quantification of MBF may also provide better risk stratification and prognostic value, and may serve as a robust and reproducible endpoint in clinical trials^6^. In accordance with other studies, in the current study a significantly lower stress MBF values in ischemic than non-ischemic myocardium have been observed. Moreover, the values of rest and stress MBF in ischemic and non-ischemic myocardium are consistent with those reported in the literature. In the studied cohort, in the non-ischemic myocardium the mean rest MBF was 1.00 ± 0.17 ml/g/min, whereas the stress MBF was 2.41 ± 0.57 ml/g/min. Recently, the largest QP analysis of healthy volunteers reported mean values of rest and stress MBF of 0.62 ± 0.13 ml/g/min and 2.24 ± 0.53 ml/g/min, respectively^36^. Other studies have shown, that in patients the mean rest and stress MBF in non-ischemic myocardium ranges between 0.9 ± 0.3 to 1.4 ± 0.4 ml/g/min and 2.3 ± 0.5 to 3.0 ± 0.8 ml/g/min, respectively^19,24,37,38^. Therefore, the proposed simplified dual-bolus protocol provides quantitative measures of rest and stress MBF in non-ischemic myocardium comparable to the dual-sequence performance. The values of rest (1.13 ± 0.18 ml/g/min) and stress (1.9 ± 0.4 ml/g/min) MBF within ischemic myocardium in the current study were also in accordance with other reports.

Sub-analysis of the CE-MARC study showed that in ischemic myocardium perfused by a significantly obstructed coronary artery (defined by quantitative coronary angiography; QCA, as ≥ 70% stenosis in any coronary artery of ≥ 2 mm diameter or ≥ 50% in the left main stem) rest and stress MBF were 1.23 ± 0.41 and 2.16 ± 0.70 ml/g/min, respectively^37^. In the study by Hsu et al., ischemic myocardium supplied by a vessel with significant obstruction (defined by QCA as ≥ 70% stenosis) had mean rest MBF 0.74 ± 0.25 ml/g/min and stress MBF 0.92 ± 0.36 ml/g/min^19^. Kotecha et al.^24^ showed, that the mean stress MBF in myocardial territories supplied by arteries with FFR ≤ 0.8 was 1.47 ± 0.48 ml/g/min, while in the study by Everaars et al.^16^, mean rest and stress MBF in myocardium supplied by non-culprit stenosed vessels with FFR ≤ 0.8 were 0.91 ± 0.29 ml/g/min and 1.90 ± 0.59 ml/min/g, respectively^24,38^. Although comparable to the study by Everaars et al.^16^, the mean value of stress MBF within ischemic myocardium in the current analysis is slightly higher when compared to the study by Kotecha et al.^24^. This discrepancy may partly be related to differences in ischemic burden, severity of obstruction, low number of patients and other factors, including pharmacotherapy, which has not been taken into account in calculating the average value of stress MBF within ischemic coronary territory. Nevertheless, the proposed workflow provides QP results consistent with values reported in the literature on dual-sequence approach.

Finally, the results suggest that the presented workflow is promising for achieving high performance in the diagnosis of obstructive CAD. The optimal cut-off value of stress MBF to detect significant coronary stenosis obtained in this study was comparable to the study by Kotecha et al.^24^ and Everaars et al.^16^ where FFR was used to define physiologically relevant obstruction^24,38^. However, Kotecha et al. showed higher AUC (0.90) and specificity (81%)^24^. One of the causes may be higher prevalence of CMD in the studied population, although this has not been routinely assessed. Moreover, it has to be noted, that one of the major limitations of perfusion CMR is the presence of the dark rim artifact. Since the dark rim artifacts are associated with loss of SI, they may give false-positive results in QP analysis and reduce the specificity and overall accuracy of the method.

To date, the diagnostic accuracy and clinical utility of pixel-wise color maps generated by the QP CMR software has not been widely studied. Biglands et al. showed in a CE-MARC sub-study, that QP CMR (ROC curve analysis of stress MBF and MPR) has high diagnostic accuracy for detecting obstructive CAD, although this was not superior to visual analysis of conventional gray-scale first-pass perfusion images^37^. However, Villa et al. have shown, that although diagnostic accuracy of QP CMR is not significantly different to visual conventional assessment performed by a level 3 CMR expert, it significantly outperforms the assessment made by level 1 and 2 readers^32^. Our results show that analysis of only color pixel-wise QP maps yield higher sensitivity to detect obstructive CAD than when combined with assessment of gray-scale images. On the other hand, the co-assessment of QP pixel maps and conventional first-pass perfusion images provides higher specificity, PPV, NPV and overall diagnostic accuracy. Presence of dark-rim artifacts may give a false-positive conclusion, which can be verified by an experienced CMR reader. It has been shown that QP-derived stress MBF values in dark rim artifact are significantly higher than in true perfusion defects resulting from obstruction in coronary artery (2.17 ± 0.61 vs. 0.95 ± 0.43 ml/min/g, p < 0.001)^39^. However, since QP CMR has not yet been widely implemented in daily clinical practice, the readers may still need to use the conventional first-pass perfusion images to verify the results of color pixel maps and make a final diagnostic conclusion. Further, larger studies will be crucial to evaluate the diagnostic performance of color pixel QP maps in a daily clinical practice with patients referred for stress perfusion CMR.

### Limitations of the study

The major limitation of the study is a small sample size and lack of inclusion of healthy volunteers. Therefore, larger validation studies are needed to confirm the presented results and establish cut-off values of stress MBF to detect obstructive CAD. Moreover, we did not perform a head-to-head comparison with the conventionally used GBCA administration schemes. In a young healthy population the blood flow may reach even 4 ml/g/min, therefore choosing the moderate infusion rate at 3 ml/s may cause a bias with underestimation of MBF in this population. In our study, the presented protocol was applied to patients suspected of having obstructive CAD, who had cardiovascular risk factors, and some of them already had a history of CAD. Therefore, a moderate infusion rate of 3 ml/s applied together with two concentrated boluses appears to be a reasonable compromise that can be implemented in daily clinical practice. After exclusion of coronary territories with LGE, the prevalence of coronary artery obstruction was relatively low. As a consequence, our approach yielded relatively low PPV. However, even based on such a small cohort, our simplified dual-bolus scanning protocol showed comparable results to other studies validating QP CMR on larger population of patients in an easy to implement approach in contemporary clinical practice. Nevertheless, larger studies involving simultaneously comprehensive assessment of coronary physiology and function of microcirculation are required to provide the precise (age- and sex-specific) cut-off values and propose a diagnostic algorithm to detect obstructive CAD and CMD using our workflow. It is also crucial to recognize the overall limitation of perfusion CMR, presence of dark rim artifact, which due to loss of SI may result in false-positive results in QP analysis and possibly affect the accuracy of this method. Finally, the fully-automated QP CMR software remains a research tool and further studies on its validation in clinical practice are warranted.

## Conclusions

This study successfully implemented a ready-to-use QP CMR workflow with simplified GBCA administration scheme which provides good image quality and holds promise for high diagnostic accuracy for detection of obstructive CAD. The used dual-bolus protocol can be easily integrated into daily clinical practice without significantly affecting the time efficiency of the CMR laboratory. Fully-automated QP software does not require manual delineation of myocardial contours and simplifies the post-processing. Finally, implementation of this approach has potential to serve as an alternative to current methods thus increasing the accessibility and ability to offer high-quality QP CMR imaging to patients suspected having CAD by a wide range of CMR laboratories.

### Supplementary Information

Supplementary Figure S1.

## Data Availability

All data generated or analyzed during this study are included in this published article and its supplementary information files.

## Abbreviations

ACE: Angiotensin converting enzyme
AIF: Arterial input function
ARB: Angiotensin receptor blockers
AUC: Area under the curve
CAD: Coronary artery disease
CCTA: Coronary computed tomography angiography
CI: Confidence interval
CMR: Cardiovascular magnetic resonance
CMD: Coronary microvascular dysfunction
Cx: Circumflex artery
FFR: Fractional flow reserve
GBCA: Gadolinium-based contrast agent
ICA: Invasive coronary angiography
IQR: Interquartile range
LAD: Left anterior descending artery
MBF: Myocardial blood flow
MI: Myocardial infarction
NPV: Negative predictive value
PCI: Percutaneous coronary intervention
PK time: Peak time
PPV: Positive predictive value
QCA: Quantitative coronary angiography
QP CMR: Quantitative perfusion cardiovascular magnetic resonance
RCA: Right coronary artery
ROC: Receiver operating characteristics
ROIs: Myocardial regions of interests
SD: Standard deviation
SI: Signal intensity
