# Supplementary material for: Clinical implementation of a fully automated quantitative perfusion cardiovascular magnetic resonance imaging workflow with a simplified dual-bolus contrast administration scheme
Source: Sci Rep. 2024 Apr 26;14:9665. doi: 10.1038/s41598-024-60503-x (PMC11053149; doi:10.1038/s41598-024-60503-x)
Supplement: Supplementary file 1 — Supplementary Figure S1. [file 41598_2024_60503_MOESM1_ESM.pdf]

Supplementary Figure S1. 5-point Likert scale for image quality assessment. CAD – coronary artery disease**.**

Measure		Answear						
QP MBF maps	Diagnosis	☐ No obstructive CAD				☐ Obstructive CAD		
Conventional grey-scale first-pass perfusion images			Points					
			1	2	3	4	5	
	Presence of artifacts	Severe artifacts	☐	☐	☐	☐	☐	No artifacts
	Presence of noise	Severe noise	☐	☐	☐	☐	☐	No noise
	Overall impression of image quality	Poor quality	☐	☐	☐	☐	☐	Excellent quality
	Certainty in diagnosis	No confidence	☐	☐	☐	☐	☐	Full confidence
Final conclusion	Diagnosis	☐ No obstructive CAD				☐ Obstructive CAD		
